# Barriers for continuous medical education: a cross-sectional questionnaire study among Danish GPs

**DOI:** 10.3399/BJGPO.2023.0228

**Published:** 2024-07-24

**Authors:** Helle Ibsen, Linda Juel Ahrenfeldt, Jesper Lykkegaard, Jens Søndergaard, Igor Švab, Niels Kristian Kjaer

**Affiliations:** 1 Research Unit for General Practice, Department of Public Health, University of Southern Denmark, Esbjerg, Denmark; 2 Research Unit for General Practice, Department of Public Health, University of Southern Denmark, Odense, Denmark; 3 Department of Family Medicine, Medical Faculty, University of Ljubljana, Ljubljana, Slovenia

**Keywords:** general practice, continuous medical education, barrier, professional competence, general practitioners

## Abstract

**Background:**

GPs’ participation in continuous medical education (CME) is essential for patient care, GPs’ wellbeing, and healthcare expenditure. However, one-quarter of Danish GPs did not use their reimbursement for CME in 2022. Knowledge of barriers for participating in CME is limited.

**Aim:**

To analyse the barriers GPs face to participation in CME, and patterns in perceived barriers.

**Design & setting:**

A cross-sectional questionnaire study design was used. The study population comprised all 3257 GPs in Denmark who, in May 2023, were registered as entitled to reimbursement for CME.

**Method:**

The response rate was *n* = 1303/3257 (40%). Based on a question about use of CME, the responders were divided into ‘frequent', ‘partial', and ‘seldom’ users. Partial and seldom users answered questions about barriers related to CME (*n* = 726). The presence of barriers was quantified, and a latent class analysis (LCA) was used to stratify GPs according to their barrier patterns.

**Results:**

The most frequent barriers were as follows: too busy (67%); fully booked courses (45%); and no substitute or locum doctor (39%). Based on the LCA, we found three distinctive patterns, clustering around the following: GPs from clinics with no tradition for CME (class 1, 17%); GPs who used time on professional work outside clinic (teaching, organisational work) (class 2, 43%); and GPs who were personally or professionally affected (class 3, 40%). Singled-handed and male GPs were slightly overrepresented among seldom users.

**Conclusion:**

We have identified barriers for CME. We found three different profiles of GPs who perceived different patterns of barriers. Identified patterns in barriers should be considered in future CME initiatives.

## How this fits in

GPs’ participation in continuous medical education (CME) is essential for the quality of patient care,^1–3^ the wellbeing of the GPs,^4^ and the level of healthcare expenditure.^5^ The value of CME is well documented and generally accepted but nevertheless some GPs do not engage in CME. Only a few studies have addressed barriers for GPs’ participation in CME. To recruit more GPs for CME, we need to understand how to make CME more achievable and attractive for GPs who are currently not engaging in CME.

## Introduction

CME and continuous professional development (CPD) describe the process where healthcare professionals engage in ongoing learning and skill development to maintain, update, and enhance their professional knowledge, skills, and competencies. In this article, we use ‘CME’ to describe this process.

### Organisation of CME for GPs

Different countries have chosen different models to ensure postgraduate education of GPs. CME can be mandatory, or it can be voluntary with the expectation that doctors have an ethical and professional obligation to undertake further education. CME can be based on public funding or rely on sponsorship from private or commercial actors. The way CME programmes are organised seems to influence the outcome.^1,6,7^ Deliberate recruitment to CME, active learner participation, and mixed interactive and didactic educational meetings improves the outcome.^1^ Funding relying on the pharmaceutical industry can bias the educational goals and topics.^6,8^ From a patient perspective, however, the most important issue is the educational outcome and the improvement of clinical care.^9^

Within Europe, CME varies from voluntary CME administered by the individual GP, to partly or mainly mandatory CME, with or without recertification.^7^

The Danish GP CME programme consists of both mandatory centrally planned activities and self-chosen voluntary activities.^10^ Both types of CME are remunerated. CME arranged by the pharmacological industry cannot be remunerated. The CME refund for a GP is approximately €6500 per year.^11^ The CME programme is based on professional integrity and trust without recertification.^10^ Despite a well-remunerated and comprehensive CME model, one-quarter of Danish GPs did not use renumeration for CME in 2022.^12^

### Barriers for participation in CME

Few studies have explored barriers for participation in CME. A study from Portugal found lack of time and bureaucracy overload as barriers for use of a digital CME platform.^13^ Two Irish studies found that main barriers for participation in mandatory CME and maintenance of professional competence was lack of protected time and financial costs of CME participation.^14,15^ Other noticeable barriers were expense of locum cover, lack of high quality and relevant CME themes, inconvenient locations, and technical difficulties.^14,15^

However, we do not know if these barriers are relevant to GPs working under the Danish model. In a former qualitative study,^16^ we uncovered 18 barriers for participation in CME. We do not know the magnitude or significance of these barriers. In a nationwide questionnaire survey in Denmark, we aim to generate quantitative data about barriers for participating in CME.

The objectives are:

1. to determine the frequency of barriers for CME;
2. to explore disparities in barriers between partial and seldom users of CME;
3. to identify profiles of GPs who experience barriers for participation in CME.

## Method

### Setting

In Denmark there is a specified specialist training scheme that lasts for 6 years to qualify as a GP.^17^ General practice plays a vital role in the Danish healthcare system.^18^ GPs are responsible for most of primary care and serve as the patient's first contact with the healthcare system.^18^ Referral from a GP is required for most office-based specialists and in- and out-patient hospital treatment.^18^ The average number of listed patients per GP is 1600.

### Study population

The study population was all GPs in Denmark who in May 2023 were registered as entitled to reimbursement for participation in CME (*n* = 3257).

### Study design

A cross-sectional questionnaire was conducted of Danish GPs’ motivation and barriers for participation in remunerated CME, and the GPs’ attitudes towards CME activities. In this article, we report data regarding barriers for participating in CME.

### Data collection

In May 2023, all registered GPs in Denmark received an email invitation with an electronically administered questionnaire. Non-responders were sent a reminder after 1 week and 2 weeks. The response ratio was calculated as responders/study population. The link to the questionnaire was personal and contained a unique serial number. Demographic data were obtained through the questionnaire and through a central database in the Danish Organisation of General Practitioners (PLO). The research group received the anonymised dataset. Data were transferred to a secure server.

### Questionnaire

The questionnaire was developed for the purpose by the research group based on literature search and interviews with 10 Danish GPs who had not used reimbursement for CME in a 2-year period.^16^ The questionnaire was evaluated for construct and content validity using an educational expert review and a pilot test.

Six skilled people reviewed the questionnaire: two with research experience, two educationalists, and two GPs with educational insight. Four GPs were strategically selected with best variance in sex, seniority as a GP, geography, and practice type. They participated in a dynamic and repeated pilot test. The questionnaire was modified in the process for clarity and comprehensibility and for content and perspective.

Responders were initially asked: ‘Do you use all your reimbursement for CME?', with response options being: ‘frequent use', ‘partial use', ‘seldom use', or ‘do not know'. ‘Partial users’ and ‘seldom users’ were asked to rate 18 barriers on a Likert scale (strongly agree, agree, disagree, strongly disagree). See Supplementary Box 1 for questions regarding barriers.

### Statistical methods

Descriptive statistics were used to examine the distribution of responses, expressed as numbers and percentages, among participants in the investigated categories ('strongly agree', ‘agree', ‘disagree', and ‘strongly disagree') in relation to the different questions about barriers. Categories with a limited number of responders were merged owing to the Danish General Data Protection Regulation (GDPR).

*P* values, used to assess differences in barriers between partial users and seldom users, were calculated using χ^2^ test or Fisher’s exact test, when the frequency in one of the cells was <5.

To investigate the representativeness of our study population, we compared baseline characteristics on sex, age, practice type, and geography in our study population with data from the PLO covering all GPs in Denmark.

To investigate whether certain barriers were more likely to be mentioned together, we employed the statistical method latent class analysis (LCA).^19^ LCA identifies patterns among selected barriers potentially revealing subgroups of GPs associated with these barriers. LCA is a statistical method employed to identify distinct subgroups within a population that share similar characteristics.^20^ The Akaike Information Criterion (AIC) and the Bayesian Information Criterion (BIC) were used to evaluate the model fit. Models with more than three latent classes were incompatible with the data. Among the models with two and three classes, the model with three classes exhibited the best fit based on both AIC and BIC. The LCA exclusively focused on barrier-related responses and were not adjusted for covariates. Nevertheless, differences in covariate distributions across the three identified classes were assessed using χ^2^ test. All analyses were conducted using Stata (version 18.0).

## Results

**Figure 1.**
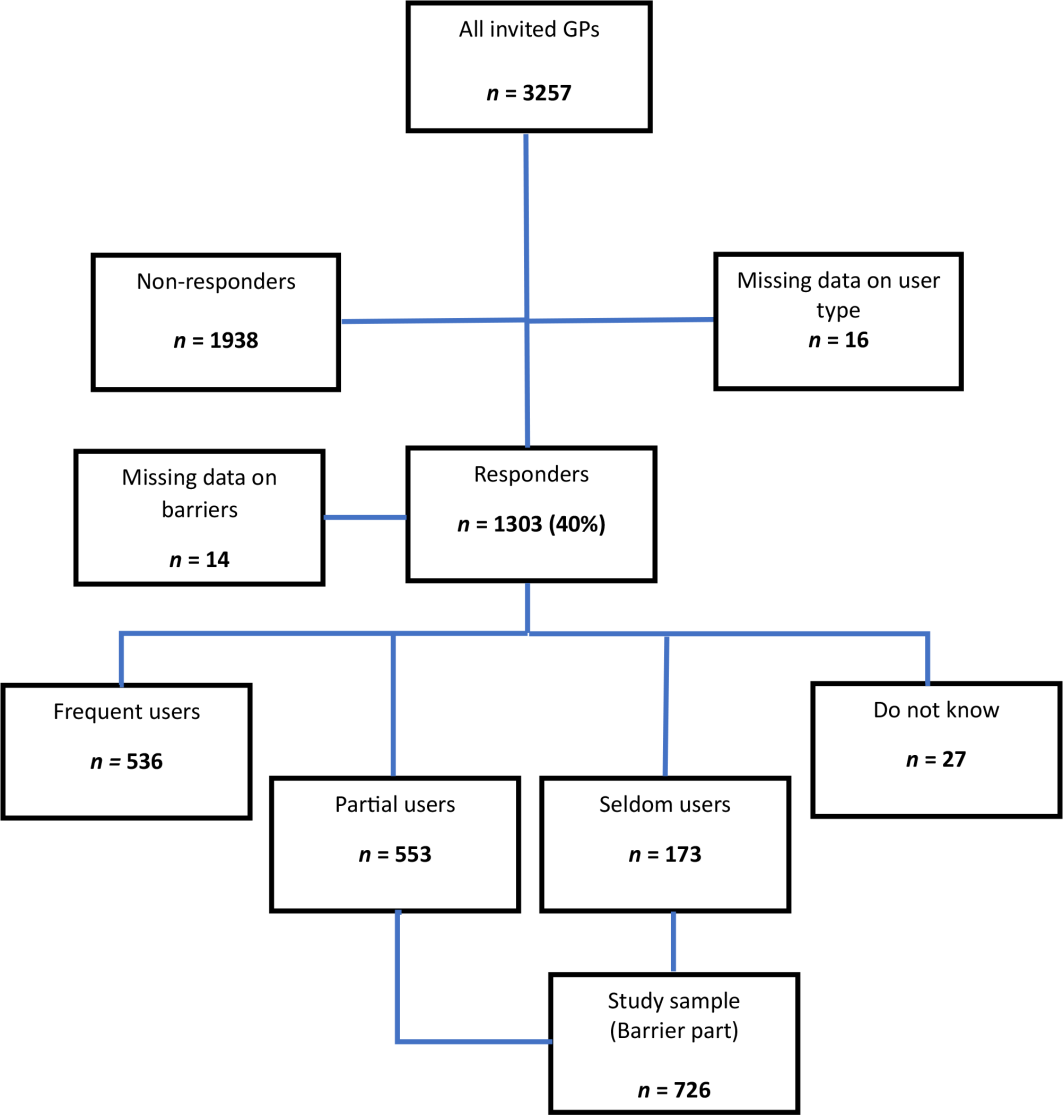
Flowchart of study population

Of the 3257 invited GPs, 1303 GPs (40%) took part in the survey. In this article, we focus on a subset of 726 GPs categorised as ‘seldom users’ (*n* = 173) and ‘partial users’ (*n* = 553), as they form the population of interest regarding barriers to CME. A flowchart of the study population is shown in Figure 1.

### Representativeness of study population

Demographic factors in the responder group (sex, age, practice type, and geographical regions in Denmark) showed a balanced distribution compared with all Danish GPs except from a larger proportion of female responders (68%) compared with overall female representation in the GP population (60%) (Supplementary Table S1).

### Association between use of reimbursement and demographic data

GPs in singled-handed practices and male GPs were overrepresented and female GPs were underrepresented among ‘seldom users'. The other demographic data were evenly distributed. Table 1 shows characteristics of our study sample ('partial users’ and ‘seldom users') compared with all responders with *P*-values for differences between groups demonstrated in Supplementary Table S2.

**Table 1.** Baseline characteristics of all responders stratified by use of continuous medical education

	Frequent users (*n* = 536, 42.5%)	Partial users (*n* = 553, 43.8%)	Seldom users (*n* = 173, 13.7%)	Total (*n* = 1262)
**Sex^a^**				
Female	386 (72.3)	370 (66.9)	98 (56.7)	854 (67.8)
Male	148 (27.7)	183 (33.1)	75 (43.4)	406 (32.2)
**Age in years, mean (SD**)	51.9 (7.6)	51.3 (8.0)	52.1 (8.7)	51.7 (8.0)
**Seniority as a GP**				
<5 years	91 (17.0)	103 (18.6)	34 (19.7)	228 (18.1)
5–15 years	229 (42.7)	268 (48.5)	86 (49.7)	583 (46.2)
>15 years	216 (40.3)	182 (32.9)	53 (30.6)	451 (35.7)
**Practice type**				
Shared practice	39 (7.3)	32 (5.8)	9 (5.2)	80 (6.3)
Partnership	410 (76.5)	408 (73.8)	95 (54.9)	913 (72.3)
Collaboration	33 (6.2)	45 (8.1)	12 (6.9)	90 (7.1)
Singled-handed	54 (10.1)	68 (12.3)	57 (33.0)	179 (14.2)

Data are *n* (%) unless stated otherwise.

SD = standard deviation.

^a^Two participants did not report their sex in the survey

### Perceived barriers

**Figure 2.**
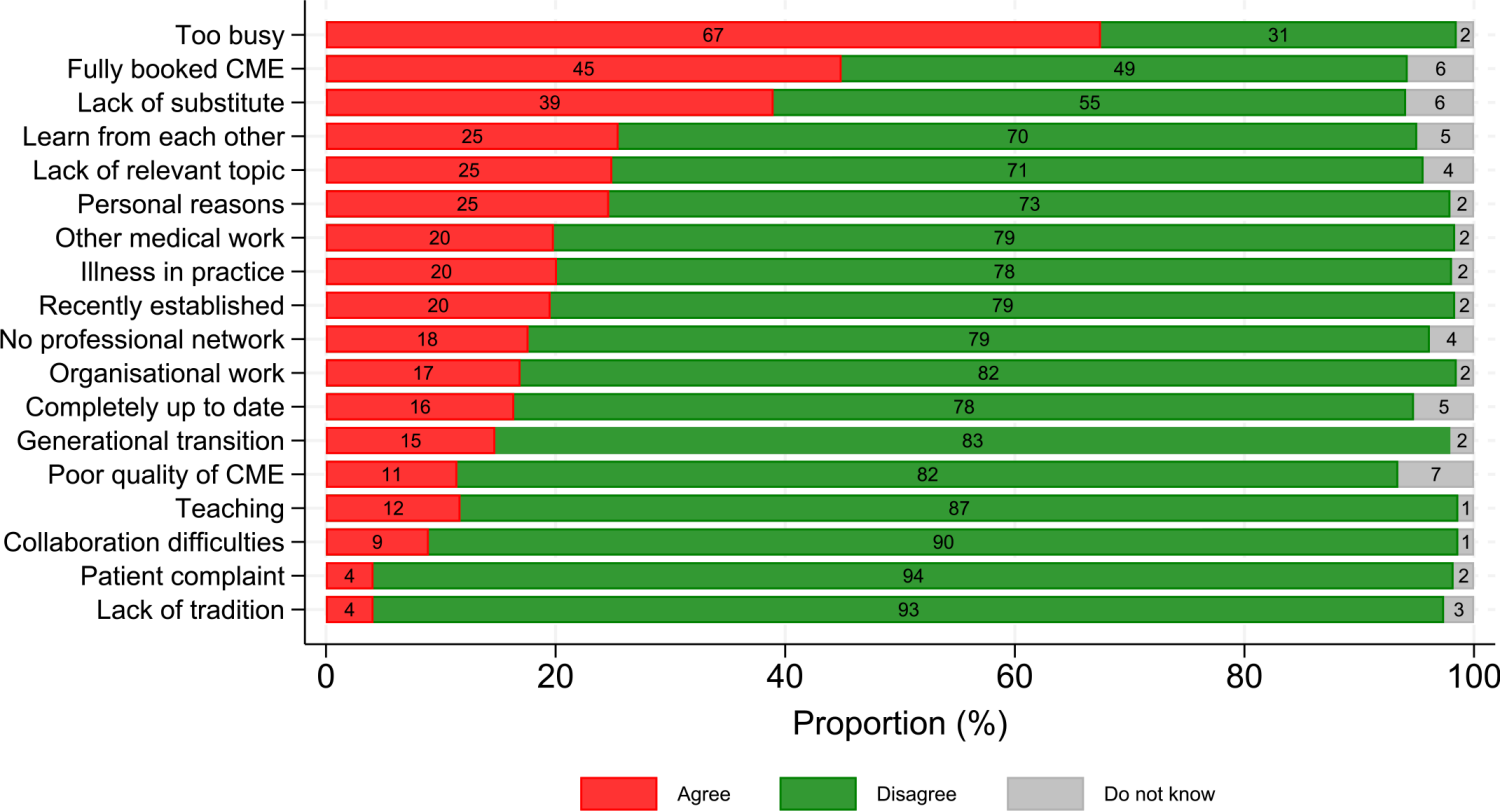
Frequency of barriers for participation in continuous medical education (CME) among GPs who are not using all their reimbursement for CME (*n* = 726)

The most frequently barrier (recognised by 67%) was being too busy. Figure 2 shows the extent to which GPs expressed agreement or disagreement within each barrier. Differences in barriers between partial and seldom users are demonstrated in Supplementary Tables S3–S6.

Fully booked courses (45%) and difficulties in finding a substitute or locum (39%) were the second and third most common barriers, whereas lack of tradition for CME, patients’ complaints, and collaboration difficulties were reported less often.

### Three characteristic patterns of barriers

The statistical method LCA revealed three subgroups (classes) among GPs. The total of 726 GPs were categorised into the following three classes: class 1 (*n*= 124, 17% of the GPs); class 2 (*n* = 310, 43% of the GPs); and class 3 (*n* = 292, 40% of the GPs).

**Figure 3.**
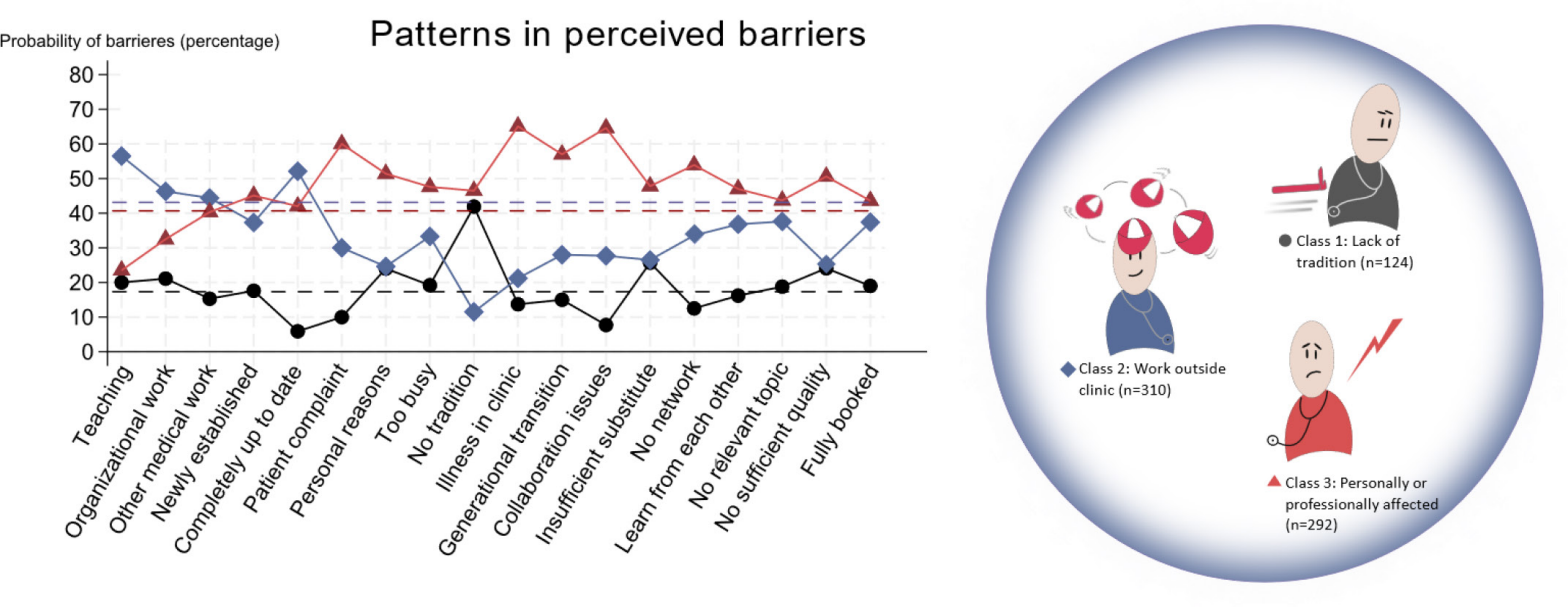
Barrier response probabilities reported by GPs with respect to the three latent classes

The distribution of the three classes and the probabilities of barrier responses reported is shown in Figure 3. When the reported barrier deviates from the dashed line in the class colour, it indicates that the barrier is selected more often or less often than expected based solely on the size of the class.

Class 1 reported ‘No tradition for CME’ as their main barrier (42%). ‘Being completely up to date’ (6%) and ‘collaboration difficulties’ (8%) were uncommon barriers in this group. The other barriers were more evenly distributed corresponding to class 1’s share of responders.

Class 2 reported ‘teaching’ (57%), and ’being completely up to date’ (52%) as frequent barriers. ‘Lack of tradition for CME’ was less frequently reported (12%). Despite activities outside clinic (teaching), class 2 had the lowest propability of reporting ‘lack of substitute’ (27%) and being ‘too busy’ (33%).

The GPs in class 3 reported ‘illness in their clinics’ (65%) ‘collaboration problems’ in their clinics (65%), ‘patients’ complaints’ (60%), or ‘generational transition’ in their clinics (57%), ‘no network’ when on courses (54%), and ‘personal reasons’ (51%), which included illness experienced by the GP themselves, more often than the two other classes.

As showed in Table 2, responders’ perceived barriers vary significantly (*P*<0.05) across the three groups in 13 of 18 barriers.

**Table 2.** The comparison of barriers for continuous medical education across latent classes (*n* = 726)

	Class 1 *n* = 124 (17.1%)	Class 2 *n* = 310 (42.7%)	Class 3 *n* = 292 (40.2%)	*P* values
Teaching	17 (20.0)	48 (56.5)	20 (23.5)	0.003*
Organisational work	26 (21.1)	57 (46.3)	40 (32.5)	0.126
Other medical work	22 (15.3)	64 (44.4)	58 (40.3)	0.797
Recently established	25 (17.6)	53 (37.3)	64 (45.1)	0.307
Completely up to date	7 (5.9)	62 (52.1)	50 (42.0)	0.001*
Patient complaint	3 (10.0)	9 (30.0)	18 (60.0)	0.097
Personal reasons	43 (24.0)	44 (24.6)	92 (51.4)	<0.001*
Too busy	94 (19.2)	163 (33.3)	233 (47.6)	<0.001*
Lack of tradition	109 (41.9)	30 (11.5)	121 (46.5)	<0.001*
Illness in practice	20 (13.7)	31 (21.2)	95 (65.1)	<0.001*
Generational transition	16 (15.0)	30 (28.0)	61 (57.0)	<0.001*
Collaboration difficulties	5 (7.7)	18 (27.7)	42 (64.6)	<0.001*
Lack of substitute or locum	119 (25.7)	123 (26.5)	222 (47.8)	<0.001*
No professional network	16 (12.5)	43 (33.6)	69 (53.9)	0.003*
Learn from each other	30 (16.2)	68 (36.8)	87 (47.0)	0.049*
Lack of relevant topic	34 (18.8)	68 (37.6)	79 (43.7)	0.235
Poor quality of CME	20 (24.1)	21 (25.3)	42 (50.6)	0.003*
Fully booked CME	62 (19.0)	122 (37.4)	142 (43.6)	0.013*

Data are *n* (%). The *P* values were computed from χ^2^ tests or Fisher’s exact test when the frequency in one of the cells was <5. Significance levels *P*<0.05 are marked in bold with asterisk.

CME = continuous medical education.

The percentages shown for the barriers do not add up to 100% for each class as they demonstrate the distribution among the three classes.

### Covariates

The responding GPs had similar sex, age, seniority as GPs, distribution of practice types, and distribution of practice owners and employed GPs between the classes.

The distribution of covariates across participants answering strongly agree and agree to barriers are demonstrated in Table 3.

**Table 3.** The covariate distribution across latent classes of situations where responders answer strongly agree or agree to barriers regarding continuous medical education (CME)

	Class 1 (*n* = 124, 17.1%)	Class 2 (*n* = 310, 42.7%)	Class 3 (*n* = 292, 40.2%)	*P*-values
**Sex^a^**				0.299
Female	82 (66.1)	190 (61.3)	196 (67.1)	
Male	42 (33.9)	120 (38.7)	96 (32.9)	
**Age in years, mean (SD**)	51.0 (7.9)	52.4 (8.4)	50.7 (8.0)	0.278
**Users of CME**				0.514
Partial users	97 (78.2)	240 (77.4)	216 (74.0)	
Seldom users	27 (21.8)	70 (22.6)	76 (26.0)	
**Seniority as a GP**				0.630
<5 years	23 (18.6)	55 (17.7)	59 (20.2)	
5–15 years	63 (50.8)	145 (46.8)	146 (50.0)	
≥16 years	38 (30.7)	110 (35.5)	87 (29.8)	
**Practice ownership**				0.664
Owner	121 (97.6)	303 (97.7)	288 (98.6)	
Employed	3 (2.4)	7 (2.3)	4 (1.4)	
**Practice type**				0.150
Shared practice	8 (6.5)	21 (6.8)	12 (4.1)	
Partnership	82 (66.1)	201 (64.8)	220 (75.3)	
Collaboration	12 (9.7)	25 (8.1)	20 (6.9)	
Singled-handed	22 (17.7)	63 (20.3)	40 (13.7)	

Data are *n* (%) unless stated otherwise. The *P*-values were computed from χ^2^ tests or Fisher’s exact test when the frequency in one of the cells was <5.

CME = continuous medical education. SD = standard deviation

^a^Two participants did not report their sex in the survey

## Discussion

### Summary

In this cross-sectional questionnaire study, uncovering GPs’ perceived barriers to CME, the most frequently reported barrier was ‘being too busy with everyday work’. All GPs reported several barriers. Combinations of selected barriers revealed three possible profiles of GPs perceiving barriers for participating in CME:

- GPs who had no tradition for CME and might need enhanced motivation;
- GPs who used time on professional work outside clinic (teaching, organisational work);
- GPs who were personally or professionally affected.

Patient complaints were reported more frequently as a barrier among the GPs who were personally or professionally affected. However, it does not necessarily imply that GPs in this subgroup receive more patient complaints. A complaint can have a negative psychological impact and may reduce GPs’ motivation for CME.

Male GPs and GPs in singled-handed practices were overrepresented among seldom users of CME.

### Strengths and limitations

All GPs in Denmark were invited to the questionary survey. The response rate of 40% was comparable with other survey studies on GPs^21,22^ but could entail selection bias. However, the study population reflected the total GP population except for a larger proportion of female GPs (68% versus 60%). This might underestimate barriers as female GPs were overrepresented among ‘frequent users’ of CME. The number of ‘seldom users’ in our study was in line with or above earlier studies.^16^

We used qualitative interviews to identify possible barriers to CME. It is a strength that the questionnaire was based on barriers defined by GPs who had not engaged in CME in a 2-year period.^16^

Using self-reported data can be a limitation if GPs are less likely to report themselves as ‘seldom users’ but the rate of responders classifying themselves as ‘seldom users’ was in line with the proportion of GPs who were not using their reimbursement in the annual report from PLO.^12^

### Comparison with existing literature

Studies from Portugal and Ireland^13–15^ found ‘lack of time’ as a main barrier, which corresponds to our finding of ‘being too busy’, as the most frequently reported barrier. Similarly, to the Irish studies,^14,15^ we found problems with locum cover as a frequent barrier.

‘Lack of quality of CME’ and ‘technical difficulties’ were seldom reported in Denmark, which differed from the Irish studies,^14,15^ whereas ‘lack of relevant themes’ was recognised in both countries. ‘Overbooked courses’ was the second most reported barrier in Denmark but to our knowledge overbooked courses are not described in other studies. Financial costs were not relevant in our study because we investigated barriers among GPs who had not used all their reimbursement.

In a former study,^16^ we found that all GPs stated ‘too busy’ but all GPs were able to point out other barriers that could explain their experience of being too busy. The barriers behind ‘too busy’ could be divided into the following three categories: barriers related to the clinic; barriers related to the CME; and personal barriers. In the present study, barriers related to CME and barriers related to the clinic were reported more often than barriers related to the individual GP. However, in our LCA, we identified a class where GPs reported personal reasons significantly more often (see Table 2). In this class, GPs expressed a higher level of being too busy compared with the other two classes. This suggests that the GP with barriers related to personal issues to a higher degree perceive themselves as overwhelmed by their workload.

We found no previous studies of underlying patterns in responders’ barrier-related responses among GPs.

We found male GPs and GPs in singled-handed practices slightly overrepresented among ‘seldom users'. A report from 2022 showed similar findings.^12^

### Implications for research and practice

Our study highlights potentials for CME organisers and health authorities. Focus on accessibility to relevant CME activities will probably support recruitment to CME.

A course that is set-up to be more adapted to busy workdays may ease the need for substitutes. In addition, facilitation of workplace learning, ‘learning from each other’, can strengthen an existing tradition.

Such a course can be appropriate with awareness of:

- newly established GPs and ongoing retirement may result in temporary disengagement with CME;
- GPs lacking a professional network may not engage with CME;
- GPs struggling with work overload and/or social challenge may need support different from traditional CME activities;
- a knowledge of profiles of GPs perceiving barriers for CME can be used to target CME to the GPs who are currently not engaging with CME.

Further research in preferred educational set-up, in attitudes to CME, and how to enhance GPs’ intrinsic professional motivation could be of interest. Additionally, there is a need to monitor barriers in future CME evaluations.

### Conclusion

We have identified barriers for CME. Some can easily be addressed while others will be far more complex to overcome. We found the following three different profiles of GPs who perceived different patterns of barriers: GPs who have no tradition for CME and may need enhanced motivation; GPs who use time on professional work outside clinic (teaching, organisational work); and GPs who are personally or professionally affected. The patterns in barriers should be considered in future CME initiatives.

## Data

The dataset relied on in this article is available from the corresponding author on reasonable request.
